# Abnormalities in early visual processes are linked to hypersociability and atypical evaluation of facial trustworthiness: An ERP study with Williams syndrome

**DOI:** 10.3758/s13415-017-0528-6

**Published:** 2017-07-06

**Authors:** Danielle M. Shore, Rowena Ng, Ursula Bellugi, Debra L. Mills

**Affiliations:** 1 Department of Experimental Psychology, University of Oxford, Tinbergen Building, 9 South Parks Road, Oxford, OX1 3UD UK; 2 Institute of Child Development, University of Minnesota, Twin Cities, 51 East River Road, Minneapolis, MN 55455 USA; 3 Laboratory for Cognitive Neuroscience, Salk Institute for Biological Studies, 10010 North Torrey Road, La Jolla, CA 92037 USA; 4 School of Psychology, Bangor University, Brigantia Building, Penrallt Road, Bangor, Gwynedd LL57 2AS UK

**Keywords:** Trustworthiness, Event-related potentials (ERP), Face perception, Williams syndrome, Approach behavior, Trust

## Abstract

Accurate assessment of trustworthiness is fundamental to successful and adaptive social behavior. Initially, people assess trustworthiness from facial appearance alone. These assessments then inform critical approach or avoid decisions. Individuals with Williams syndrome (WS) exhibit a heightened social drive, especially toward strangers. This study investigated the temporal dynamics of facial trustworthiness evaluation in neurotypic adults (TD) and individuals with WS. We examined whether differences in neural activity during trustworthiness evaluation may explain increased approach motivation in WS compared to TD individuals. Event-related potentials were recorded while participants appraised faces previously rated as trustworthy or untrustworthy. TD participants showed increased sensitivity to untrustworthy faces within the first 65–90 ms, indexed by the negative-going rise of the P1 onset (oP1). The amplitude of the oP1 difference to untrustworthy minus trustworthy faces was correlated with lower approachability scores. In contrast, participants with WS showed increased N170 amplitudes to trustworthy faces. The N170 difference to low–high-trust faces was correlated with low approachability in TD and high approachability in WS. The findings suggest that hypersociability associated with WS may arise from abnormalities in the timing and organization of early visual brain activity during trustworthiness evaluation. More generally, the study provides support for the hypothesis that impairments in low-level perceptual processes can have a cascading effect on social cognition.

Trust is elemental for successful navigation of day-to-day social environments. Researchers have argued that the ability to accurately assess trustworthiness is crucial to survival (Cosmides & Tooby, 2000). Trustworthiness judgments are important for informing approach or avoid decisions (Oosterhof & Todorov, 2008; Winston, Strange, O’Doherty, & Dolan, 2002) and also drive interpersonal trust decisions (Schlicht, Shimojo, Camerer, Battaglia & Nakayama, 2010; van ’t Wout & Sanfey, 2008). Many social environments involve strangers, for whom the only trust information initially available is their facial appearance. Accordingly, people make trust judgments from facial appearance alone (Zebrowitz & Montepare, 2008). These facial judgments are made quickly and reliably (Bar, Neta & Lintz, 2006; Willis & Todorov, 2006). Although these impressions are not always accurate (Olivola & Todorov, 2010), evidence suggests that trustworthiness evaluations may be made automatically, even when trustworthiness is not relevant to the current task (Engell, Haxby & Todorov, 2007; Shore, Mills, & Dishion, 2013). This research supports the idea that humans have developed a specialized neural mechanism that facilitates fast and automatic trustworthiness evaluation (Marzi, Righi, Ottonello, Cincotta, & Viggiano, 2012).

Functional brain imaging studies of typically developing adults and adolescents show increased activation of the amygdala to untrustworthy compared to trustworthy faces (Haas, Ishak, Anderson, & Filkowski, 2015; Mattavelli, Andrews, Asghar, Towler, & Young, 2012; Todorov, Mende-Siedlecki, & Dotsch, 2013; Rule, Krendl, Ivcevic, & Ambady, 2013). Individual variability in amygdala activity is also correlated with ratings of trustworthiness for faces (Haas et al., 2015). Investigation of the time course of trustworthiness evaluation using event-related potentials suggests that this mechanism likely begins in early visual processing of face stimuli (Dzhelyova, Perrett, & Jentzsch, 2012). The amygdala receives information from the occipital cortex and fusiform gyrus in a feed-forward cortical network (Fairhall & Ishai, 2007). As such, these early visual processes may be fundamental for adaptive decision making. But the time course of these processes and how they interact with sociability are not well understood. The present study examines the temporal dynamics of trust evaluation in individuals with Williams syndrome (WS), a genetic disorder linked to both visuospatial deficits and hypersociability.

When first viewing a face, people make multiple trait inferences, which stem from categorizations on two primary dimensions: dominance and trustworthiness (Todorov, Said, Engell, & Oosterhof, 2008). Research suggests that trustworthiness judgments may reflect the overgeneralization of features that look like emotional expressions, that is, the similarity of a neutral face to a smiling or angry expression (Adams, Nelson, Soto, Hess, & Kleck, 2012; Engell, Todorov, & Haxby, 2010; Oosterhof & Todorov, 2009; Said, Sebe, & Todorov, 2009). Neutral faces that resemble a smile are evaluated as trustworthy, whereas a neutral face that resembles an angry expression is evaluated as untrustworthy (Said et al., 2009). Functionally, these trust evaluations shape expectations about a partner’s likely behavior and, thus, when other information is limited, guide decision making until more, and more reliable, information becomes available (Cosmides & Tooby, 2000; Frith & Frith, 1999). In accord, these trustworthiness evaluations inform approach/avoidance behavior (Oosterhof & Todorov, 2008).

When evaluating information from faces, threat-related stimuli should receive preferential processing, as it is adaptive to attend to potentially dangerous or harmful stimuli in the environment (Öhman, 1986). In accordance with the threat-detection hypothesis, studies on the neural time course of facial trustworthiness evaluation find that early attentional processing is biased toward threatening faces (Schupp et al., 2004), including untrustworthy faces (Marzi et al., 2012; Yang, Qi, Ding, & Song, 2011). Further, research shows that negative appearance-based attributions drive trust-related behaviors such as voting (Spezio et al., 2008). As trust judgments are crucial for successful navigation of the social world (Cosmides & Tooby, 2000), this study tested the importance of early visual processing for adaptive trust based decision making. This study aimed to address this question by assessing the temporal dynamics of facial trustworthiness evaluation in individuals with WS.

WS is a rare neurodevelopmental disorder characterized by a greater tendency to approach strangers (Doyle, Bellugi, Korenberg & Graham, 2004; Jones et al., 2000; Zitzer‐Comfort, Doyle, Masataka, Korenberg, & Bellugi, 2007) leading to increased social vulnerability (Fisher, 2014). It results from a hemizygous deletion of 26 to 28 genes on chromosome 7q11.23 (Korenberg et al., 2000). The sociocognitive profiles in WS are mirrored in the neuroanatomical profiles of the WS brain (Reiss et al., 2004). Compensating for an overall reduction in brain size, areas linked to emotion and the relative strengths in face identity processing are proportionally larger than expected with increased size of the amygdala (Capitao et al., 2011) and the fusiform face area (FFA; Golarai et al., 2010; although see Meyer-Lindenberg et al., 2004). In contrast, deficits in visuospatial skills are associated with volumetric and morphological abnormalities, including curtailment of the occipital and parietal lobes (Reiss et al., 2004), abnormal gyrification in these areas (Gaser et al., 2006), and abnormal cell size and cell packing density in primary visual cortex (Galaburda, Holinger, Bellugi, & Sherman, 2002).

Previous research shows increased approach behaviors toward untrustworthy faces (Martens, Hasinski, Andridge, & Cunningham, 2012), and atypical assessment of stranger-danger scenarios in individuals with WS (Riby, Kirk, Hanley, & Riby, 2014). Compared to typically developing adults (TD), individuals with WS exhibit atypical reasoning about the trustworthiness of others, such that they are less able to discriminate between trustworthy and untrustworthy individuals (Ng, Fillet, DeWitt, Heyman, & Bellugi, 2015). They also show a bias toward faces in general (Dodd, Porter, Peters, & Rapee, 2010) and positive faces specifically (Frigerio et al., 2006).

Recognition of facial identities is a relative strength in WS, with normal or near normal performance on identity recognition (Bellugi, Lichtenberger, Jones, Lai, & St. George, 2000). In contrast, recognition of emotional expressions is poor in WS (Plesa-Skwerer, Faja, Schofield, Verbalis, & Tager-Flusberg, 2006; Levy, Pluber, & Bentin, 2011). Performance on tests such as the DANVA (Nowicki & Duke, 1994) show performance similar to mental-age-matched or age-and-IQ-matched controls. Participants with WS show better recognition for happy expressions, with particular deficits in recognition of fearful, sad, and angry expressions (Santos, Silva, Rosset, & Deruelle, 2010).

Eckert and colleagues (2006) suggest that hypersociability in WS may be linked to abnormalities in early visual perceptual processes. Accordingly, they argue that abnormal sensory processing has a cascading effect on higher level processes such as social cognition and language. Individuals with WS show atypical face perception and neural processing of facial identity (Grice et al., 2001; Mills et al., 2000; Mills et al. 2013), positive and negative emotional expressions (Haas et al., 2009), and 3-D faces (Bernardino, Castelhano, Farivar, Silva, & Castelo-Branco, 2013) within the first 200 ms of viewing a face. Specifically, individuals with WS show decreased neural activity to fearful expressions and increased neural activity to happy expressions (see also Meyer-Lindenberg et al., 2005). To understand the extent to which these early visual attention and perceptual processes are key to adaptive functioning and trustworthiness assessments, this study tested early neural processing of trustworthiness information from faces in WS and TD participants. This study used the event-related potential (ERP) technique, as its excellent temporal resolution is particularly well suited to studying the time course of brain activity in social perception (Amodio, Bartholow, & Ito, 2014). The temporal sequence of ERP components elicited when viewing faces were recorded and assessed for components previously found to relate to face and trust information processing (Marzi et al., 2012; Yang et al., 2011).

According to the threat-detection hypothesis, early neural mechanisms may be specialized for threat detection. ERPs reveal sensitivity to untrustworthy compared to trustworthy faces within the first 100 ms of viewing a face, reflected in increased amplitudes of the C1 (Yang et al., 2011) and P1 (Marzi et al., 2012) components. The C1, an early visual ERP component, reflects bottom-up processing of motivationally salient visual features (Pourtois, Grandjean, Sander, & Vuilleumier, 2004; Stolarova, Keil, & Moratti, 2006). Accordingly, previous research with typically developing adults has found larger C1 amplitudes for threat-related stimuli (Pourtois et al., 2004; Yang et al., 2011), indicating enhanced perceptual processing of those stimuli. The C1 is believed to originate in the striate cortex, peaking within the 40 to 90-ms window over occipital-parietal sites. A defining characteristic of the C1 is that it reverses polarity over the upper and lower visual fields, shows a focal distribution over posterior occipital regions, and is often not observed for faces presented in the central visual field (Rauss, Schwartz, & Pourtois, 2011). The stimuli in the Yang et al. (2011) study were centrally presented, and the distribution of the component identified as the C1 showed a central distribution. Therefore, it is not clear if the ERP elicited in that study was the C1 or the onset phase of the P1.

The P1, a face-sensitive positivity around 100 ms, has also been shown to be enhanced for untrustworthy faces (Marzi et al., 2012). It is well established that the amplitude and latency of the P1 are sensitive to emotional expressions (e.g., Batty & Taylor, 2003; Meaux, Roux, & Batty, 2014; Pourtois et al., 2004; Pourtois et al., 2005; Williams, Palmer, Liddell, Le Song, & Gordon, 2006). Like the C1, the amplitude of the P1 is increased to fearful or threat-related facial expressions. In contrast to the C1, the scalp distribution of the P1 varies across studies, from broadly distributed over frontotemporal regions to a focal distribution over lateral occipital regions, depending on the specific task and stimuli. Source localization studies suggest activation of the anterior cingulate and extrastriate occipital regions (Santesso, Meuret, Hofmann, Mueller, Ratner, Roesch, & Pizzagalli, 2008; Williams et al., 2006). Williams and colleagues (2006) postulated that enhancement of the early positivity to fearful expressions reflects an automatic alerting mechanism to potential threat. Supporting the evolutionary importance of trustworthiness evaluations, we predict the ERP components within the first 100 ms will be modulated according to facial trustworthiness information for the TD group. Additionally, if hypersociability in WS is linked to abnormalities in perceptual processes for detecting threat-related visual cues, we predict the early enhancement of ERPs to untrustworthy faces within this range will be absent in adults with WS.

While early processing involves increased perceptual attention to untrustworthy faces, some later processes show biases toward trustworthy faces as potentially rewarding stimuli. Research shows that trustworthy faces are rewarding, capture attention in general, and receive preferential attentional processing (Singer, Kiebel, Winston, Dolan, & Frith, 2004; Shore & Heerey, 2013). In previous ERP research, increased activity to trustworthy relative to untrustworthy faces was observed around 150 ms over frontal sites, called the early frontal positivity (EFP; Marzi et al., 2012; Rudoy & Paller, 2009). The EFP is thought to reflect the reward values of preferred stimuli. We predicted that both WS and TD participants would show an increased EFP to trustworthy faces.

An ERP component selectively sensitive to faces over other types of stimuli is the N170. The N170 indexes the level of structural encoding of a stimulus, especially faces (Bentin, Allison, Puce, Perez, & McCarthy, 1996). The N170 was found to be larger over the right hemisphere to untrustworthy male faces and trustworthy female faces (Dzhelyova et al., 2012). This suggests that adaptive facial stimuli may receive facilitated structural encoding, linked to social categorization processes during early structural processing. However, the N170 is not consistently modulated by emotional expression or trustworthiness (e.g., Holmes, Vuilleumier, & Eimer, 2003). When the amplitude does differ, the N170 is larger to threat-related stimuli such as fearful faces. The amplitude of the N170 is also modulated by emotional skills in TD participants (Meaux et al., 2014). Recent evidence suggests that it is influenced by the complexity of the judgment being made; for example, more complex judgments about faces result in larger N170 amplitudes (Marzi et al., 2012). Thus, higher levels of structural encoding may be reflected in the N170 for more in-depth face evaluations.

Another ERP component sensitive to trustworthiness is the late positive potential (LPP), a slower positive deflection found on centroparietal sites from around 300 ms. The LPP is postulated to arise from reciprocal activation of prefrontal and occipital-parietal regions, indicating combined bottom-up and top-down functions (Cuthbert, Schupp, Bradley, Birbaumer, & Lang, 2000; Moratti, Saugar, & Strange, 2011). It reflects emotional and motivational processing, with larger (more positive) amplitudes to more arousing emotional stimuli (Hajcak, MacNamara & Olvet, 2010; Keil et al., 2002; Schupp et al., 2004), especially faces (Ferri, Weinberg, & Hajcak, 2012). For face stimuli specifically, LPP amplitudes are larger for negative emotional faces (Smith, Weinberg, Moran, & Hajcak, 2013), including untrustworthy faces (Marzi et al., 2012; Yang et al., 2011), when the likelihood of a face being trustworthy or untrustworthy is equal. However, in a study investigating emotion face processing in WS individuals a larger positivity was found in the LPP time window for happy faces (Haas et al., 2009). As trustworthy faces have features that look like happy emotional expressions (e.g., Oosterhof & Todorov, 2009), they may be more arousing stimuli and thus evoke a larger LPP in individuals with WS.

The present study examined whether the heightened social approach behavior in WS individuals may be explained by differences in early visual attention and perceptual processing of face stimuli. Specifically, this study tested the idea that those with WS may show atypical patterns in the timing and organization of brain activity when processing trustworthiness information from faces, compared to TD individuals. Using a simple rating task, both behavioral and electrophysiological responses to trustworthy and untrustworthy face stimuli were recorded. Behaviorally, we predicted that WS participants would make more trusting responses than TD adults. Further, in accord with previous research findings, we predicted that WS individuals would show less discrimination in approach responses between high- and low-trust faces compared to the TD group (Ng et al., 2015). We expected TD participants to make more avoid responses to low-trust faces and more approach responses to high-trust faces.

Based on the threat-detection hypothesis and previous research, we predicted that TD adults would show increased early neural activity to untrustworthy versus trustworthy faces within the first 100 ms, but participants with WS would not. We also predicted that, consistent with our previous ERP studies (Haas et al., 2009; Mills et al., 2000; Mills et al. 2013), the WS group would show increased activity to trustworthy over untrustworthy faces linked to later attentional and evaluative processes, indexed by the EFP and LPP. For TD participants we expected to see the same pattern of increased activity to trustworthy faces in the EFP; however, in line with previous studies, we expected a larger LPP for untrustworthy faces. For the N170, we expected no difference in TD participants. For WS participants, we expected to see larger N170 amplitudes for more complex judgments, in this case, to untrustworthy compared to trustworthy faces.

In addition, we predicted that modulations in ERP amplitudes linked to evaluation of trustworthiness would be positively correlated with behavioral measures of approachability in WS participants (EFP and LPP). We also expected a positive correlation between the N170 and approachability measures. Specifically, we predicted larger N170 amplitudes would be associated with lower approachability ratings. For TD participants only, we predicted that the early neural activity (prior to 100 ms) would correlate with approachability such that larger C1/onset P1 responses would relate to lower ratings of approachability. Finally, in TD participants, the EFP and LPP would show positive correlations with approachability measures.

## Method

### Power analysis

Our main hypothesis test of interest was the interaction between trustworthiness (high or low; within-subjects factor) and group (TD or WS; between-subjects factor). Therefore, to guide sample size, an a priori power analysis using G*Power 3 (Faul, Erdfelder, Lang, & Buchner, 2007), indicated that a total sample of 34 people would be needed to detect medium effects (*f* = 0.25) with 80% power for a repeated-measures ANOVA within–between interaction with α = .05. To detect a medium effect (f = 0.25) with 90% power required a total sample of 46 people. Therefore, we targeted a minimum sample size of 34 but aimed to recruit as close as possible to 46, recognizing potential constraints for participant numbers due to the use of a clinical population. Previous studies investigating ERPs to facial trustworthiness evaluation, and ERPs in WS participants support an expectation of a medium effect size (Fishman, Yam, Bellugi, Lincoln, & Mills, 2011; Marzi et al., 2012; Yang et al., 2011).

### Participants

A total of 41 adults participated in the current study, consisting of 20 WS individuals and 21 TD comparison individuals. Participants were recruited nationally and from the local community at the Salk Institute. Table 1 shows the demographic characteristics of participants. Fluorescence in situ hybridization (FISH) probes for elastin (ELN) were employed to confirm the genetic diagnosis of WS (Korenberg et al., 2000). All WS participants exhibited the clinical phenotype of the syndrome, based on caregivers’ responses on a WS Diagnostic Score Sheet (American Academy of Pediatrics, Committee on Genetics, 2001). Exclusionary criteria included a history of neurological insult, psychiatric diagnoses, and/or central nervous system disorders. Participants were native English speakers and had normal or corrected-to-normal vision. Participants gave written consent, and caregivers or conservators of WS individuals provided an additional informed written assent. The Institutional Review Board at the Salk Institute approved experimental procedures. Handedness was assessed by asking participants and their caregivers, and was verified using the Edinburgh Handedness Inventory (Oldfield, 1971).

**Table 1:** Participant characteristics

	Mean (*SD*)[Range]	
	Williams syndrome (*N* = 20)	Typical development (*N* = 21)
Age (years)	32.98 (10.35) [19.00–56.88]	27.26 (6.55) [18.10–43.09]
Sex	7F	13F
Handedness	17R, 3L	17R, 4L
VIQ	66.26 (7.05)	98.06 (14.42)
PIQ	65.00 (5.44)	96.00 (14.21)
FIQ	73.37 (9.18)	99.05 (16.14)
SISQ-global	5.31 (1.32, *n* = 18)	4.38 (.912, *n* = 15)

SISQ = Salk Institute Sociability Scale

### Stimuli

A total of 100 headshots (50 female, 50 male) with a neutral expression from the FERET Database were edited to remove hair, accessories (e.g., earrings), and clothing (Phillips, Wechsler, Huang, & Rauss, 1998). Faces were prerated for trustworthiness by 20 undergraduate college students on a 7-point Likert scale (1 = *extremely untrustworthy*, 7 = *extremely trustworthy*; for more information on prerating of faces, see He, 2011). The 50 highest rated faces (25 male, 25 female; *M* = 5.6) were classified as high-trust faces, while the 50 lowest rated (25 male, 25 female; *M* = 4.4) were classified as low-trust faces.

### Procedures

After completing consent/assent procedures, participants were seated in a quiet room that was customized to reduce noise interference, and the EEG cap was fitted.

**Fig. 1:**
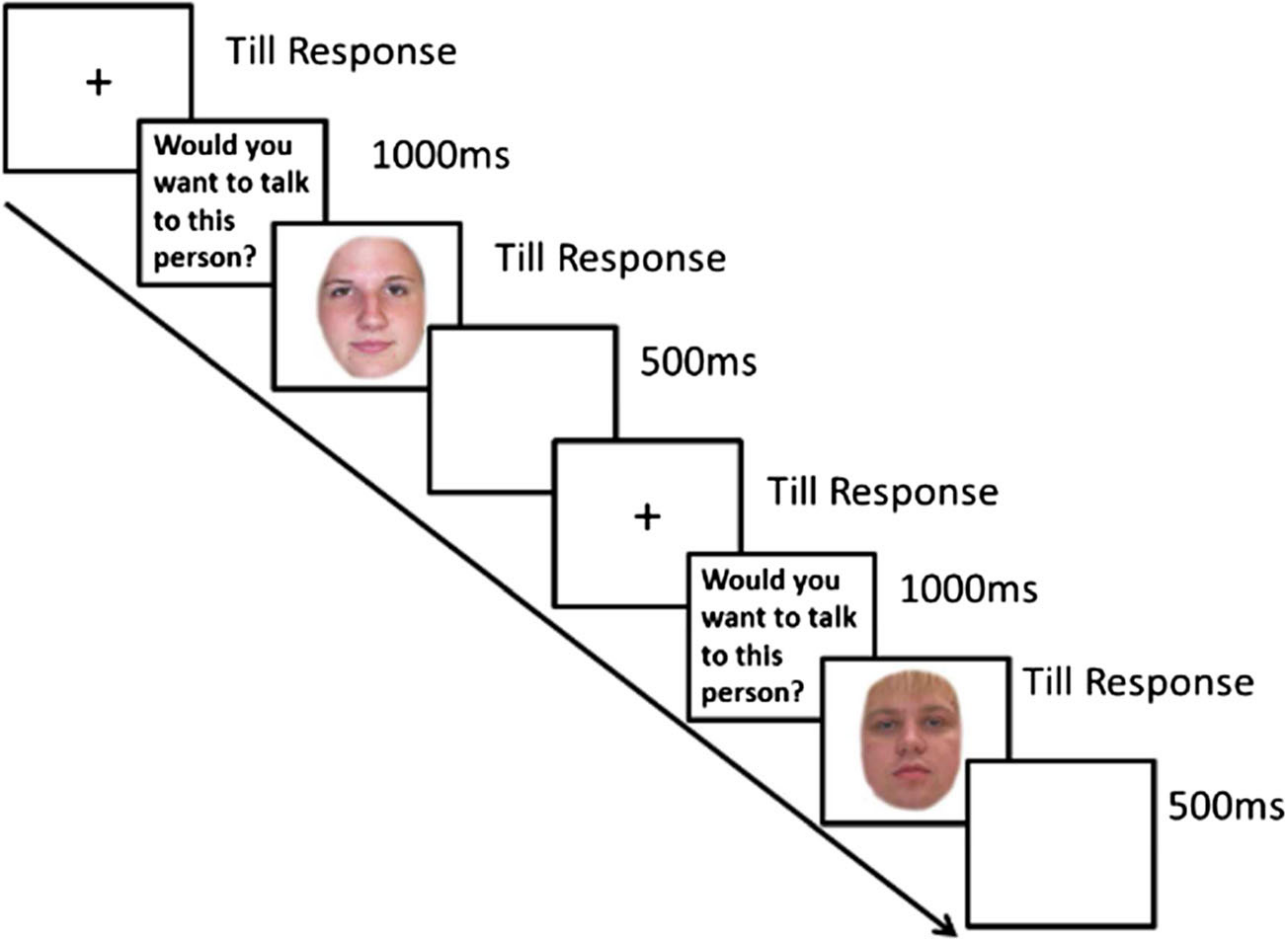
Trial timeline. Faces were 320 × 240 pixels and presented in a 50% square in the center of the screen, positioned approximately 60 cm away from the participant

Participants first saw instructions to respond to the question, “Would you want to talk to this person?” for each face in the block, using a button-press to indicate yes, maybe, or no.^1^ One hundred face stimuli were presented in the same randomized order. Prior to each face, participants viewed a fixation cross until they pressed a key to start the trial and the question (1,000 ms). After each face, there was an interstimulus interval (500 ms). Faces were presented in the center of the screen until participants made a response (see Fig. 1). The task was programmed and presented using E-Prime (Psychology Software Tools Inc., Pittsburgh, PA, USA).

### Cognitive and sociability assessment

To assess cognitive functioning for both TD and WS participants, the Wechsler Adult Intelligence Scale–Third Edition (WAIS-III; Wechsler, 1997) and the Wechsler Abbreviated Scale of Intelligence (WASI; Wechsler, 1999) were administered. TD individuals only completed the WASI, whereas WS individuals were administered the WAIS-III. As shown in Table 1, TD participants outperformed the WS group across verbal IQ, performance IQ, and full-scale IQ (*t*s > 8.59, *p*s < .001).^2^

The Salk Institute Sociability Scale (SISQ; Jones et al., 2000) was used as a convergent measure of group differences in social drive. The SISQ questionnaire assesses different aspects of increased social drive in WS, including willingness to approach familiar and unfamiliar people and behavior in social situations (Doyle et al., 2004; Jones et al., 2000). The inventory consists of questions designed to gather qualitative data regarding participants’ social and emotional behaviors, and 12 items assessing three subscales (Sociability to Strangers, Sociability to Familiar Individuals, and Social-Emotionality), which together yield an overall (global) Sociability score. Of the 12 items, four assess Social-Emotionality (e.g., How likely is your child to comment on the emotional state of other individuals? 1 = *not likely to comment*, 7 = *extremely likely to comment*), five evaluate Sociability to Strangers (e.g., My child would spontaneously greet or approach an unfamiliar peer; 1 = *very rarely*, 7 = *very often*), and three evaluate Sociability to Familiar Individuals (e.g., My child would spontaneously greet or approach a member of his/her immediate family; 1 =*very rarely*, 7 = *very often*). For more psychometric information regarding the SISQ, see Doyle et al., (2004) and Zitzer-Comfort et al. (2007). Higher SISQ Sociability scores were observed for the WS than for the TD participants for the overall Sociability measure (see Table 1), as well as for the Sociability to Strangers subscale (*t*s > 2.4, *p*s < .02). 3

### EEG recording

Electrophysiological data was recorded from a 64-channel Ag/AgCl electrode HydroCel Geodesic Sensor Net (Geodesic EEG System 300, Electrical Geodesic Inc, Eugene, OR) with an online reference at the vertex (Cz). The EEG was sampled at 250 Hz, with a bandpass of .1–100 Hz. The electrooculogram was recorded from over and under the left eye to detect blinks and vertical eye movements, and from the right outer canthus to detect horizontal eye movements. Off-line data was rereferenced to average activity of the left and right mastoids and low-pass filtered at 30 Hz. Event-related potentials (ERP) for face displays were segmented over an epoch of 1,100 ms (starting 100 ms prior to the presentation of the face), using a 100 ms prestimulus baseline. The ERPs for each trial were checked for artifact, and trials containing eye movements or deflections exceeding +/-200 mV were removed. Separate ERP average waveforms were computed for trustworthy and untrustworthy faces, for the TD and WS participants. For both trustworthy and untrustworthy faces, the number of trials retained for analysis (out of 50) for TD participants was significantly higher (*M* = 36.90, *SD* = 8.26; and *M* = 37.04, *SD* = 7.80, respectively) than for WS participants (*M* = 26.45, *SD* = 12.39; and *M* = 26.60, *SD* = 13.30, respectively), *F*(1, 39) = 10.75, *p* = .002, η^2^_p_ = .22. We appreciate that artifact correction methods could have been used to minimize group differences in the number of trials retained for analysis. Artifact correction, using ICA or subtraction methods, can introduce distortions over frontal regions and thus still disproportionately affect averages with uneven number of trials (Luck, 2014). This difference in the number of trials in the grand average waveforms means that main effects of group should be interpreted with some caution, as the signal-to-noise ratio might differ. However, there were no differences in the number of trials per condition for within-group comparisons.

## Results

### Behavioral data

An average approachability rating was calculated for each participant by computing their average response to the question “Would you want to talk to this person” (*yes* = 3, *maybe* = 2, and *no* = 1) separately for the 50 high- and 50 low-trust faces. These scores were entered into a repeated-measures ANOVA with trust (high or low) as a within-subjects factor and group (TD or WS) as a between-subjects factor. As expected, there was a main effect of trust, *F*(1, 39) = 19.88, *p* < .001, η_p_^2^ = .34, with high-trust faces being rated as more approachable (*M* = 2.04, *SD* = 0.38) than the low-trust faces (*M* = 1.84, *SD* = 0.44). The interaction with group was not significant, suggesting that, contrary to prediction, WS participants were not significantly more trusting than the TD group, *F*(1, 39) = 0.41, *p* = .52, η_p_^2^ = .01.

**Fig. 2:**
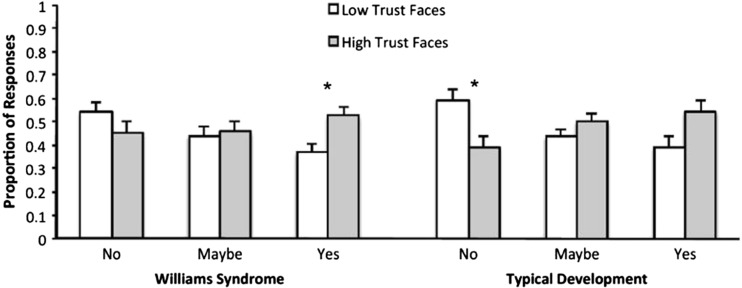
Proportion of approach, maybe, and avoid responses for high- and low-trust faces by TD and WS participants (*error bars* indicate +/- 1 *SE*). *p < .05

Given our hypothesis that WS participants would show less discrimination in their responses to trustworthy and untrustworthy faces, we computed the proportion of approach (yes), maybe, and avoid (no) responses to high- and low-trust faces separately for each participant. For example, if a participant made 40 approach responses, 10 to low-trust faces and 30 to high-trust faces, the proportion of approach responses would be 0.25 and 0.75, respectively. A repeated-measures ANOVA, with trust (high or low) as a within-subjects factor and group (TD or WS) as a between-subjects factor, found a higher proportion of approach responses to high-trust (*M* = 0.54, *SD* = 0.22) compared to low-trust faces (*M* = 0.39, *SD* = 0.19), *F*(1, 39) = 9.38, *p* = .004, η_p_^2^ = .19. No other main effects or interactions were significant (all *F*s < 1, all *p*s > .53). To assess within-group patterns of approach ratings toward trustworthy or untrustworthy faces, these proportions were entered into paired *t* tests. As shown in Fig. 2, participants with WS did not discriminate between high- and low-trust faces for *no* responses (*p* > .19). However, WS participants made *yes* responses more frequently to high- compared to low-trust faces, *t*(19) = 3.22, *p* = .004. In contrast, TD adults responded *no* to low-trust faces more than high-trust faces, *t*(20) = 2.32, *p* = .03, but did not respond *yes* to high-trust faces more frequently than low-trust faces, *t*(20) = 1.82, *p* = .08. This suggests that while TD individuals show the expected discrimination in their avoid responses, WS participants demonstrate atypical approach discrimination. Specifically, while WS participants approach trustworthy faces more than untrustworthy faces, they do not discriminate trustworthiness when making avoid responses.

Reaction times were also entered into a repeated-measures ANOVA, with trust (low or high) as a within-subjects factor and group (WS or TD) as a between-subjects factor. No main effects or interactions were observed (*F*s < 1.14, *p*s > .29), with similar average reaction times for both TD participants (*M* = 1673.96, *SD* = 910.48) and WS participants (*M* = 1621.06, *SD* = 1281.16).

### ERP data

Components of interest were chosen from the ERP waveforms based on previous research and visual inspection of the data. The components of interest were the onset phase of the P1, EFP, N170, and LPP. Each component was quantified by analyzing the mean amplitude within a specific time window centered around the maximum peak of the component. The electrode sites for each component were selected based on previous research and the topographical distribution of ERP effects. For each analysis, an average was calculated for the selected electrode cluster for each condition. ERP data were all analyzed using repeated-measures ANOVAs, with trustworthiness (high or low) as within-subjects factors and group (TD or WS) as a between-subjects factor. There were no significant correlations of ERP effects with age, gender, or IQ when groups were considered separately or together; therefore, these factors were not included in analyses.

**Fig. 3:**
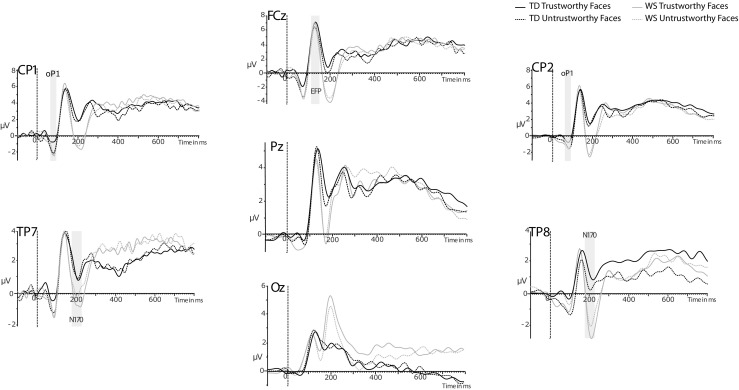
Grand average ERP waveforms for TD and WS participants to low-trust and high-trust faces

**Fig. 4:**
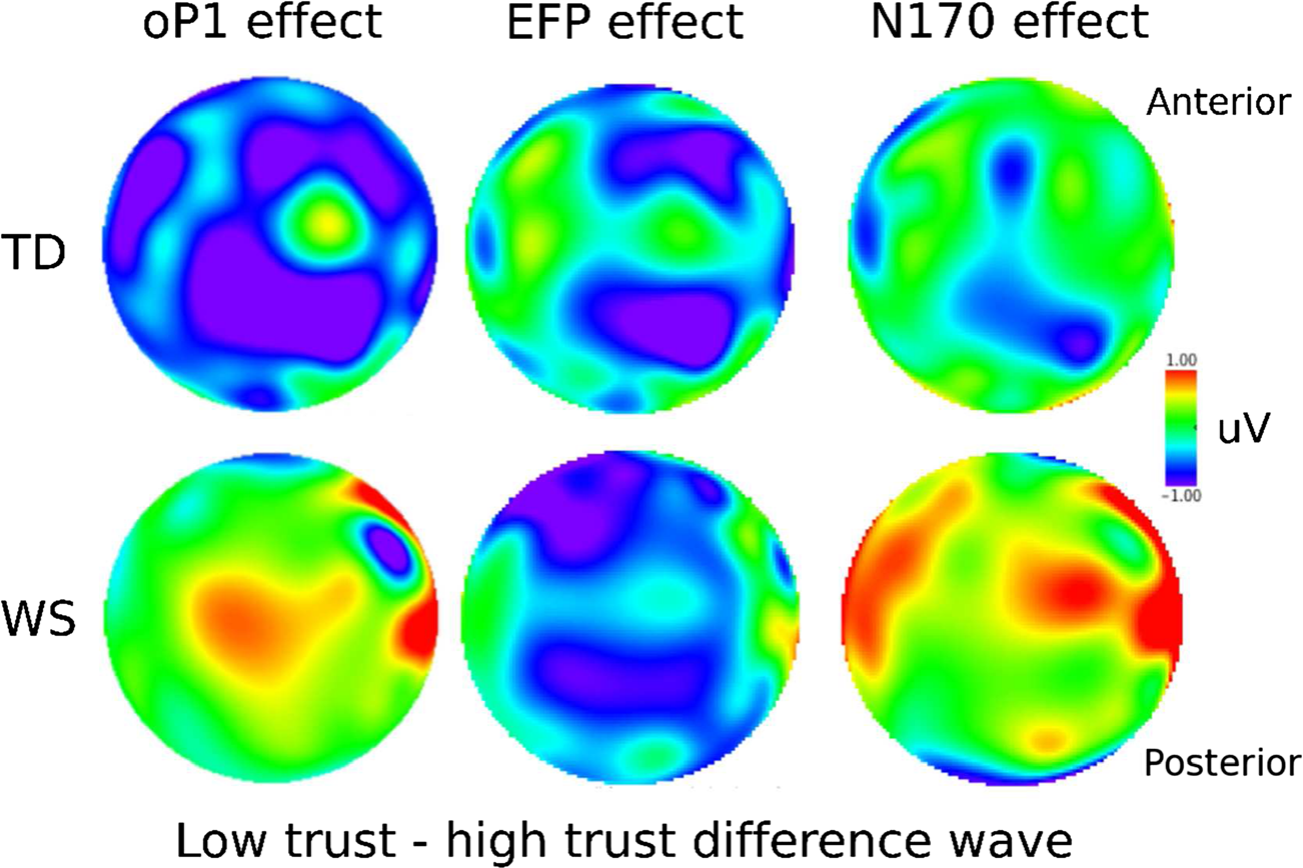
Topographical maps for oP1, EFP, and N170 effects. Maps were created using the low trust–high trust difference wave. (Color figure online)

As predicted, untrustworthy faces elicited an early component in the 65 to 90-ms window poststimulus onset. This component was negative going in amplitude and preceded the P1. Therefore, we refer to the component as the negative-going onset phase of the P1 (oP1). The distribution of the oP1 was centroposterior (see Figs. 3 and 4).^4^ In line with previous studies, the average mean amplitude across six electrodes (CP1, CP2, P1, Pz, P2, POz) was entered into an ANOVA. There was no main effect of trustworthiness or group (*F*s < 2.75, *p*s > .11). The trustworthiness by group interaction was significant, *F*(1, 39) = 4.39, *p* = .04, η_p_^2^ = .10. As predicted, TD participants had enhanced oP1 amplitudes for low-trust faces compared to high-trust faces, *t*(20) = 2.58, *p* = .02; whereas, oP1 amplitudes to high-trust and low-trust faces in WS participants did not differ, *t*(19) = −0.29, *p* = .77.

The EFP component was measured in the 130 to 170-ms window as the average mean amplitude across two frontocentral electrode sites (Fz and FCz; see Fig. 4). For the EFP component, there was a main effect of trustworthiness, *F*(1, 39) = 4.03, *p* = .05, η_p_^2^ = .09, with a larger positivity elicited by high-trust faces than low-trust faces in both groups (see Figs. 3 and 4). This suggests that both TD and WS participants are processing trustworthy faces as preferable or more rewarding than untrustworthy faces. There were no other main effects or interactions (all *F*s < 0.92, *p*s >.40).

To assess if TD and WS participants differed in their structural processing of faces, an analysis was performed on the N170. The N170 component was quantified as the mean amplitudes in the 180 to 220-ms window and analyzed at electrode sites (TP7, TP8, T7, T8; see Fig. 3). As previous studies support expectations for laterality effects in the N170, the average of the electrodes on the left (TP7 and T7) and right (TP8 and T8) hemispheres were calculated, and laterality was included in the ANOVA. For the N170 component there was a significant interaction of trustworthiness and group, *F*(1, 39) = 5.01, *p* = .03, η_p_^2^ = .11. For TD participants, N170 amplitudes did not differ between high-trust and low-trust faces, *t*(20) = 0.57, *p* = .57, while WS participants had greater N170 amplitudes to high-trust faces compared to low-trust faces, *t*(19) = −2.44, *p* = .03. This suggests that WS participants may perform enhanced structural encoding for trustworthy faces compared to untrustworthy faces. There was also a main effect of laterality, *F*(1, 39) = 4.85, *p* = .03, η_p_^2^ = .11, such that the N170 was larger in amplitude on the right compared to the left hemisphere. In addition, there was a significant interaction of laterality and group, *F*(1, 39) = 4.45, *p* = .04, η_p_^2^ = .10. Simple main effects analysis showed that there was no difference in N170 amplitude in the left hemisphere between WS and TD (*p* = .18); however, the N170 was more negative in WS in the right hemisphere compared to TD (*p* < .001). Finally, there was a main effect of group, *F*(1, 39 = 12.77, *p* = .001, η_p_^2^ = .25, with more negative-going amplitudes in WS than in TD. No other main effects or interactions were significant (all *F*s > 3.87, all *p*s > .07).

The LPP was measured using the average mean amplitudes from the time window (300–500 ms) across nine centroparietal electrode sites (C1, C2, C3, C4, CP1, CP2, Pz, P1, P2). Results of the ANOVA showed no significant main effects or interactions (all *F*s < 0.95, *p*s > .34). The predicted main effect of trustworthiness was not found.

### Correlations of ERPs and behavioral data

Each participant’s average approachability ratings from the ERP task were used to correlate a behavioral measure of approachability with the observed ERP effects above. The average approachability ratings from the ERP task correlated with the SISQ subscale across both groups for SISQ Social-Emotionality, *r*(38) = .35, *p* = .03. For the TD group only, the SISQ global measure significantly correlated with average approachability ratings from the ERP task, *r*(15) = .53, *p* = .04. These findings show that the behavioral measure from the ERP task is related to real-life social behaviors. Because recent SISQ measures were not available for all participants, the average approachability ratings from each participant were used for correlations with ERP effects.

Mean ERP scores for the oP1, EFP, and N170, were calculated by averaging the component amplitudes across the sites used in the analyses noted above. Effect (difference) scores were calculated by subtracting the mean ERP scores between high and low trust conditions. Correlations of ERP effects with approachability scores were calculated separately for each group.

**Fig. 5:**
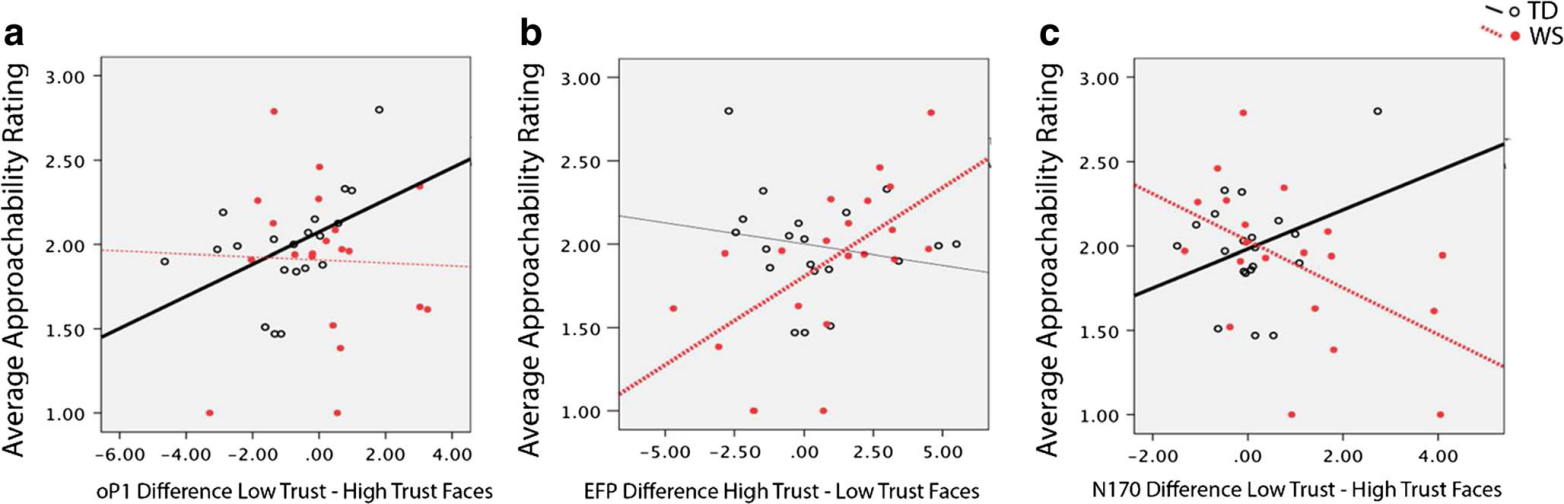
Correlations between ERP effects for the oP1, EFP, and N170 components and approachability ratings for TD and WS participants. *Bold lines* indicate significant correlations. (Color figure online)

The oP1 ERP difference effect to low-trust–high-trust faces correlated with the average approachability score for the TD group, *r*(21) = .50, *p* = .02, but not the WS group, *r*(20) = −.03, *p* = .89. Because the oP1 is a negative-going waveform, the positive correlation indicated that the larger (i.e., more negative going) the oP1 difference to low-trust minus high-trust faces, the lower the approachability score for the TD participants (see Fig. 5a).

The EFP difference effect showed that a larger amplitude EFP to high-trust compared to low-trust faces was associated with higher approachability scores for the WS participants, *r*(20) = .60, *p* = .005, but not the TD group, *r*(21) = −.19, *p* = .43 (see Fig. 5b).

The N170 difference to low-trust–high-trust faces showed opposite correlations for the two groups. For the TD group, a larger (i.e., more negative going), N170 to low-trust–high-trust faces was correlated with lower approachability, *r*(21) = .46, *p* = .04. In contrast, for participants with WS, the N170 untrustworthy–trustworthy effect was correlated with higher approachability, *r*(20) = −.50, *p* = .02 (see Fig. 5c).

## Discussion

The findings showed that when processing faces, TD adults displayed the expected ERP effects for greater perceptual attention orienting to untrustworthy faces within the first 100 ms (oP1), followed by later increased activity to trustworthy faces (EFP) and equal structural encoding of faces (N170). Heighted activity to untrustworthy faces was associated with decreased approachability in TD adults. Conversely, WS individuals did not differentiate between perceptual processing of trustworthy versus untrustworthy faces in the oP1 and showed heightened structural processing of trustworthy faces in the EFP and N170. Although both groups showed larger EFP amplitudes to trustworthy faces, the EFP effect was correlated with increased approachability ratings only for the participants with WS. Also for individuals with WS, the lack of increased N170 activity to untrustworthy faces was linked to increased approachability ratings. Thus, both the timing and organization of neural activity linked to evaluation of trustworthiness is atypical in WS. Together these combined behavioral and electrophysiological results are consistent with the hypothesis that early visual and attentional processing biases toward untrustworthy faces are disrupted in individuals with WS. Concomitantly, structural encoding of trustworthy faces in WS individuals appears to be heightened. To our knowledge, this is the first study to show this double dissociation, that brain activity within the first 100 ms of viewing a face is linked to opposite patterns of sociability in neurotypic adults and adults with an atypical neuro-social profile.

The current study replicated the finding by Yang et al. (2011) with TD adults, showing heightened activity to untrustworthy faces within the first 100 ms. Yang and colleagues (2011) identified this as the C1 component. In the present study, we labeled this early negativity as the onset phase of the P1. The oP1, like the C1 in the Yang study, was elicited to centrally presented faces and displayed a central distribution. What was novel about our study is that increased activity to untrustworthy faces in this time window (65–90 ms) was correlated with avoidance ratings for TD adults, but not in WS. In other studies, the P1, which peaks slightly later in the waveform (~130 ms), amplitudes are also modulated by threat detection, increased to fearful expressions (Pourtois et al., 2004) and untrustworthy male faces (Marzi et al., 2012). Source localization studies suggest that the C1 reflects the initial perceptual processing of a stimulus in the primary visual cortex, whereas the P1 has an extrastriate origin and is sensitive to attention (Pourtois et al., 2004). Future research is needed to determine if the oP1 observed here reflects activity in striate or extrastriate regions, is sensitive to visual field presentation as is the C1, or is modulated from attentional systems acting on visual cortex. However, the temporal window suggests that the functional significance of the component reflects sensory-attentional processes.

The findings from the present study demonstrate that TD individuals show an adaptive response, with a larger oP1 for threat related (untrustworthy) faces, and correlations of the oP1 effect associated with a general tendency to avoid approaching unfamiliar faces. In contrast, WS participants do not show enhanced early processing of untrustworthy faces in the oP1. The lack of an oP1 effect or correlation of the oP1 effect with avoidance of untrustworthy faces in WS is consistent with the hypothesis that abnormalities in visual-attentional processes contribute to the tendency to indiscriminately approach strangers. One interpretation of this finding is that individuals with WS are less sensitive to visual stimuli in general. The visual cortex is proportionally smaller in WS, along with abnormalities in cell packing density and cortical thickness (Galaburda et al., 2002; Reiss et al., 2004; Thompson et al. 2005). However, fMRI-based retinotopic mapping shows that area V1 is functionally normal in response to rotating hemicircles and expanding rings (Olsen, et al., 2009). Also, for participants with WS, the oP1 to all faces is as large as the oP1 observed for the TD group to untrustworthy faces. This is important because it shows group differences are not simply due to less activity in visual cortex in the WS group. Nor can the results be explained in terms of latency jitter, which would result in smaller amplitudes for the group with more latency jitter. Another interpretation is that the oP1 in persons with WS resembles the low-trust response of the TD group. This might suggest an increased avoidance response to all faces, but there was no correlation of the oP1 with approachability in the WS group. Group differences in absolute amplitude are difficult to interpret. What is important is that the oP1 shows differential activity linked to trustworthiness evaluation for the TD group, whereas it does not in the WS group. We propose that in WS, the visual cortex fails to compute statistical regularities that indicate adaptive information, like trustworthiness. This is line with research that suggests the importance of low-level perceptual categorization processes for social cognition (e.g., Eckert et al., 2006). A recent computational model showed that using statistical regularities in low-level visual cues, like edge detection, are sufficient to discriminate higher level categories, such as animals versus man-made objects (Perrinet & Bednar, 2015). Additionally, research with other types of visual stimuli, such as animals versus man-made tools, is needed to examine the specificity of this effect to threat related stimuli.

Consistent with previous findings (Marzi et al., 2012; Rudoy & Paller, 2009), the initial bias toward untrustworthy faces in the TD group was followed by a larger anterior positivity, EFP, from 130 to 170 ms to trustworthy faces for both TD and WS groups. This shows that although individuals with WS can discriminate trustworthy from untrustworthy faces behaviorally, the time point at which neural activity diverges for trustworthy versus untrustworthy faces is later than in TD adults. Specifically, differences between the neural response to trustworthy and untrustworthy faces arise after 65 ms in TD, but not until 170 ms for WS (N170). This suggests that individuals with WS and TD adults are most likely using different brain regions to discriminate facial trustworthiness. Further, the moderate to large correlation with average approachability for participants with WS suggests that increased brain activity to trustworthy faces within the first 170 ms may be fundamental to approach behavior. Other research shows the amplitude of the EFP is modulated by emotional expression (Eimer, Kiss, & Holmes, 2008). Emotional expression is often linked to trustworthiness ratings for faces, with trustworthy faces being linked to happy expressions and untrustworthy linked to anger (Adams et al., 2012; Engell et al., 2010; Oosterhof & Todorov, 2009; Said et al., 2009). The alternative hypothesis that the EFP and other components are modulated by emotional expression rather than trust is discussed below.

In the present study, only the WS group showed N170 amplitude differences to trustworthy and untrustworthy faces. Also, the N170 was larger for participants with WS than in the TD group. We have not observed a larger N170 in previous face studies (Haas et al., 2009; Mills et al., 2000), nor are we aware of any other published work showing this effect. Thus, it is unlikely there is a general tendency for the N170 to be larger in this group. The N170 to faces has been localized to the FFA (Halgren, Raij, Marinkovic, Jousmäki, & Hari, 2000). While we did not perform source localization in this study to confirm the source of the N170 component, evidence across several different face paradigms find the FFA to be the source (Deffke et al., 2007; Halgren et al., 2000; Herrmann, Ehlis, Muehlberger, & Fallgatter, 2005; Rossion, Joyce, Cottrell, & Tarr, 2003). In WS, the FFA is proportionally twice the size observed in TD adults, relative to both total brain area and the fusiform gyrus (Golarai et al., 2010). In that study, FFA volume was correlated with performance on a face identity task for participants with WS. In TD individuals, the FFA has been shown to be sensitive to structural regularities (e.g., symmetry) in face-like stimuli (Caldara & Seghier, 2009). It is possible that in WS, the FFA acts as a compensatory mechanism for detecting structural regularities associated with trustworthiness. We suggest that what was accomplished earlier in the visual stream, as indexed by the oP1 to untrustworthy faces in TD participants, may instead be processed a few ms later in the EFP, and in different brain areas such as the FFA, as indexed by the N170, in WS.

The extant literature shows mixed sensitivity of the N170 to trustworthiness and emotion in neurotypic populations. In studies that do show an effect on the N170 in TD adults, the N170 is larger to negative expressions (Blau, Maurer, Tottenham, & McCandliss, 2007; Righart & de Gelder, 2008). In contrast to our original hypothesis, participants with WS showed continued increased brain activity to trustworthy faces. One possible explanation for this finding is that judging a trustworthy face is more complex for individuals with WS. As previous research suggests N170 amplitudes are influence by judgment complexity (Marzi et al., 2012), this result may reflect the fact that WS participants are engaging in higher levels of structural encoding to judge whether a trustworthy face should be trusted; whereas, untrustworthy faces require less cognitive resources to distrust.

Although only the WS group showed a larger N170 to trustworthiness effect, both groups showed a correlation of N170 amplitude differences with approachability ratings. For the TD group, the correlation was in the expected direction, with a larger magnitude N170 to untrustworthy faces associated with increase avoidance. In contrast, for WS, the same N170 difference to untrustworthy–trustworthy faces was correlated with increased approachability ratings. That is, for individuals with WS, increased approachability was linked to diminished activity to untrustworthy faces. Decreased ERP activity to threat and increased activity to trust is reminiscent of the findings in the ERP/fMRI (Haas et al., 2009) study showing decreased activity to fear and increased activity to happy expressions in WS. In a related study, Haas and colleagues (2010) also showed that the decreased amygdala activity to fearful emotional expressions was correlated with increased approachability ratings.

Another hypothesis for how individuals make trustworthiness judgments is that they reflect overgeneralization of facial characteristics that resemble emotional expressions (e.g., Montepare & Dobbish, 2003; Oosterhof & Todorov, 2009). This finding suggests that WS participants may be uniquely doing this for trustworthy faces but not for untrustworthy faces, which may contribute to their proclivity to approach such stimuli (e.g., Frigerio et al., 2006). Some evidence suggests that individuals higher in empathy show greater N170 responses to facial expressions compared to emotionally neutral faces (Choi et al., 2014); however, the N170 does not differ according to the displayed expression but reflects emotion face processing in general. Individuals with WS show high levels of empathy (Hoeft et al., 2009; Mervis & Klein-Tasman, 2000; Meyer-Lindenberg, Mervis, & Berman, 2006) paired with a bias toward positive faces (Frigerio et al., 2006). Given that trustworthiness judgments from faces are linked to valence judgments (Said et al., 2009; Todorov et al., 2008), this suggests that, unlike TD individuals, WS participants may be attending to trustworthy faces as a positive expressive stimulus.

The explanation that the ERP findings related to trust in the present study could be explained solely by emotional expression is unlikely. Haas et al. (2009) used a combined ERP/fMRI approach in which participants with WS, age-matched TD individuals, and developmentally delayed controls viewed happy, fearful, neutral, and scrambled faces, in a gender discrimination task. In that study, the time course of fMRI-based activation of decreased amygdala activity to fear and increased activity to happy expressions was substantially later than observed in the present study (i.e., decreased N200 to fear and increased P300-500 to happy expressions). Also, in that study, emotional expression did not modulate the amplitude of the N170 in WS participants, developmentally delayed, or TD controls. Nor was there an early effect in the first 100 ms to fearful compared to happy or neutral expressions. This indicates that nonidentical systems may be involved in evaluation of emotional expressions and trustworthiness. Indeed, as other researchers have posited, it is likely that humans have a specialized neural mechanism for evaluating trustworthiness (Marzi et al., 2012).

Contrary to prediction, no effects were observed for the LPP component for either group. One explanation is that as the LPP is linked to arousal (e.g., Cuthbert et al., 2000), the lack of LPP differences may reflect that there were no differences in arousal between the high-trust and low-trust faces used in this study. Previous studies that have found LPP effects related to trustworthiness (Marzi et al., 2012; Yang et al., 2011) have used computer-generated faces from bald males displaying neutral facial expression. The present study used consensus preratings from more naturalistic stimuli. It is possible that the stimuli used to construct categories for trustworthy and untrustworthy faces might explain the lack of an LPP in the present study. Previous research has shown that decisions regarding the structural features used to rate trustworthiness are highly consistent across individuals (Bar et al., 2006). A large body of research is devoted to assessing the structural features used to assess trustworthy faces (Todorov et al., 2008). A future direction would be to systematically manipulate each of the features to determine which ERP components are modulated by each feature or set of features.

One limitation of the present study is that participants responded to the question of whether they would talk to the person in the picture. This simple question was used as a measure of sociability consistent with previous research with WS (Bellugi et al., 2000; Fishman et al., 2011; Järvinen-Pasley et al., 2010; Martens, Wilson, Dudgeon, & Reutens, 2009) and was similar to the Adolphs approachability task (see Bellugi, Adolphs, Cassady, & Chiles, 1999). Unlike the question “Would you trust this person,” the talk question was understood by the WS participants (see also Järvinen, Ng, & Bellugi, 2015). Approachability ratings are highly correlated with trustworthiness evaluations (Oosterhof & Torodov, 2008) and reflect how one perceives the trustworthiness of an individual. It may be that while trust is a key element of the decision to talk to someone, facial trustworthiness information may not be the only attribute that is evaluated for this decision. However, faces varying in levels of trustworthiness modulate brain activity in the amygdala, putamen, and other areas linked to evaluation of trustworthiness, even if the participants are not engaged in a trustworthiness evaluation task (Engell et al., 2007; Todorov, Baron, & Oosterhof, 2008). While the use of this question, rather than a more direct approach/avoid question (e.g., “Would you approach this person”), may engender other facial characteristic evaluations, the correlations between approachability ratings and ERP components linked to perception of trustworthiness suggest that approachability is captured by the use of this question. In addition, the low-trust faces used in this paradigm were rated as averagely trustworthy (around the midpoint of the rating scale). This may have made the task more difficult for individuals with WS, as previous research suggests they are less able to discriminate trustworthy from untrustworthy faces (Ng et al., 2015). However, the results from this study show that WS participants were able to discriminate between high- and low-trust faces when making trust responses.

A second potential limitation is that ERPs were time locked to faces previously rated as trustworthy or untrustworthy by a separate sample of undergraduate students. Another approach would have been to time lock ERPs to each participant’s own rating for how likely they were to talk to the person. This would provide an individualized measure of ERPs rated as yes, maybe, and no; however, as we expected abnormal trust responses from participants with WS, preratings were used to ensure that ERPs were recorded to identical stimuli across groups, and the number of trials per condition were equal. Moreover, evaluations of facial trustworthiness across several participants have proven more reliable than individual participant’s judgments (Engell et al., 2007). As individuals with WS are typically less discriminatory in rating responses, we wanted to examine how their neural responses to different face types differed from TD participants, to examine whether the differences in face processing may relate to approach behavior. Future studies could assess differences in the neural processing of judged trustworthy compared to untrustworthy faces in WS individuals, to elucidate how they make these distinctions.

Behaviorally, while TD adults selectively indicated they would approach high-trust faces and avoid low-trust faces, WS participants showed atypical discrimination when discerning avoidance responses. While WS participants wanted to approach trustworthy-looking strangers over those who appeared less trustworthy, TD adults selectively wanted to avoid untrustworthy strangers. Those with WS did not distinguish facial trustworthiness when avoiding others. This may indicate that the cognitive processes involved in deciding to trust someone may differ from those involved in distrust.

Together, the findings provide support for the hypothesis that real-life hypersociability in WS may arise from low-level visual processes linked to abnormal perceptual processing of others faces (Eckert et al., 2006; Järvinen-Pasley et al., 2010). Previous research has linked hypersociability in WS with decreased amygdala activation to fearful expressions (e.g., Haas et al., 2010; Meyer-Lindenberg et al., 2005). This is the first study to provide neurobiological evidence that links early visual cortex activity during evaluation of trustworthiness with social approach and avoidance in both WS and TD populations. The lack of an oP1 effect suggests that visual cortex abnormalities fail to detect low-level structural indices of threat in untrustworthy faces in WS. Models of visual processing of facial expressions suggest that activity in visual cortex precedes activation in amygdala and other areas related to processing emotion and trustworthiness (Fairhall & Ishai, 2007; Pessoa & Adolphs, 2010). Based on our findings, we suggest that the source of the abnormal amygdala function observed to negative facial expression in WS results, at least in part, from impaired or impoverished input from visual cortex, as indicated by the oP1 and N170 effects. In turn, the absence, or degradation of, neural activity indicating potential threat may be interpreted by the amygdala as positive information. Continued enhanced brain activity to trustworthy faces supports the hypothesis that impairments in early low-level perceptual processing of faces can have a cascading effect on social cognition. If this interpretation is correct, the findings have implications for designing treatments. Training the brain to pick up on statistical regularities in facial features associated with untrustworthiness might reduce the tendency to trust or walk away with a stranger. An interesting alternative is that increased attention to faces exhibited by people with WS could in turn change the way visual areas process social stimuli. Research examining personality traits such as extroversion show enhanced allocation of attention to social stimuli (Fishman, Ng, Bellugi, 2012). However, the time course of activation was much later (i.e., the P300) than in the present study. To determine a causal role for the direction of the effect would require a different methodology, such as TMS. Future research using this paradigm with populations showing contrasting patterns of perceptual and sociocognitive processing (e.g., autism) will further enhance our understanding of how early perceptual processes influence higher social cognitive processes.

## Footnotes

1 This question was used as pilot data and a previous behavioral study (Ng et al., 2015) showed WS participants did not fully understand the abstract question, “Would you trust this person?”

2 Two TD adults and one WS participant did not complete the IQ test, as they were unable to return for a subsequent testing session.

3 The SISQ was not fully completed for all participants, and as some participants only completed some subscales, their SISQ-global scores could not be calculated.

4 In the TD participants, the distribution of the oP1 effect was similar to that of the C1 found by Yang and colleagues (2011).
